# Dissociable effects of oxycodone on behavior, calcium transient activity, and excitability of dorsolateral striatal neurons

**DOI:** 10.3389/fncir.2022.983323

**Published:** 2022-10-26

**Authors:** Joshua Barry, Katerina D. Oikonomou, Allison Peng, Daniel Yu, Chenyi Yang, Peyman Golshani, Christopher J. Evans, Michael S. Levine, Carlos Cepeda

**Affiliations:** ^1^Intellectual and Developmental Disabilities Research Center (IDDRC), Jane and Terry Semel Institute for Neuroscience and Human Behavior, University of California, Los Angeles, Los Angeles, CA, United States; ^2^Department of Neurology, University of California, Los Angeles, Los Angeles, CA, United States; ^3^Department of Psychiatry and Biobehavioral Sciences, David Geffen School of Medicine at University of California, Los Angeles, Los Angeles, CA, United States; ^4^West Los Angeles VA Medical Center, Los Angeles, CA, United States; ^5^Brain Research Institute, University of California, Los Angeles, Los Angeles, CA, United States

**Keywords:** oxycodone, striatum, cerebral cortex, Miniscopes, locomotion, Ca^2+^ transients, electrophysiology

## Abstract

Opioids are the most common medications for moderate to severe pain. Unfortunately, they also have addictive properties that have precipitated opioid misuse and the opioid epidemic. In the present study, we examined the effects of acute administration of oxycodone, a μ-opioid receptor (MOR) agonist, on Ca^2+^ transient activity of medium-sized spiny neurons (MSNs) in freely moving animals. Ca^2+^ imaging of MSNs in dopamine D1-Cre mice (expressing Cre predominantly in the direct pathway) or adenosine A2A-Cre mice (expressing Cre predominantly in the indirect pathway) was obtained with the aid of miniaturized microscopes (Miniscopes) and a genetically encoded Cre-dependent Ca^2+^ indicator (GCaMP6f). Systemic injections of oxycodone (3 mg/kg) increased locomotor activity yet, paradoxically, reduced concomitantly the number of active MSNs. The frequency of Ca^2+^ transients was significantly reduced in MSNs from A2A-Cre mice but not in those from D1-Cre mice. For comparative purposes, a separate group of mice was injected with a non-Cre dependent Ca^2+^ indicator in the cerebral cortex and the effects of the opioid also were tested. In contrast to MSNs, the frequency of Ca^2+^ transients in cortical pyramidal neurons was significantly increased by oxycodone administration. Additional electrophysiological studies in brain slices confirmed generalized inhibitory effects of oxycodone on MSNs, including membrane hyperpolarization, reduced excitability, and decreased frequency of spontaneous excitatory and inhibitory postsynaptic currents. These results demonstrate a dissociation between locomotion and striatal MSN activity after acute administration of oxycodone.

## Introduction

Opioids, in particular oxycodone, are currently the treatment of choice for moderate to severe pain (Kibaly et al., 2021). However, the increase in the number of oxycodone prescriptions has led to an opioid epidemic and a consequent increase in the number of opioid-related deaths (Vearrier and Grundmann, 2021). An intimate knowledge of the mechanisms of opioid actions in the central nervous system is a prerequisite for the design of rational therapies against opioid addiction.

Pain relief by opioids is mediated primarily by the μ-opioid receptor (MOR) subclass of G-protein-coupled receptors (GPCRs). In the case of oxycodone, this opioid also binds, albeit with much lower affinity, to κ- and δ-opioid receptors (Beardsley et al., 2004). Binding to MORs inhibits adenylate cyclase and cAMP production (Sesena et al., 2014). One of the main effects of opioids is to inhibit neurotransmitter release by blocking voltage-gated P/Q-type (Cav2.1) and N-type (Cav2.2) calcium (Ca^2+^) channels (Eggermann et al., 2011). Inhibition of Cav2.1 and Cav2.2 channels *via* GPCRs is determined by direct interaction of Gβγ with the channel pore formed by the α1A or α1B Cav2.1 or 2.2 subunit (Zamponi and Currie, 2013). In addition, opioids open G-protein-coupled inwardly rectifying potassium channels resulting in hyperpolarization and overall reduction of neuronal excitability (Law et al., 2000).

μ-opioid receptors are abundant in various brain regions, including the ventromedial (VMS) and the dorsolateral striatum (DLS). However, little is known about potentially differential effects of opioids on direct and indirect pathway medium-sized spiny neurons (MSNs), which can be identified by the selective expression of dopamine D1 and D2 or, more accurately, adenosine A2A receptors. In a previous study, we demonstrated differential effects of DAMGO, a selective MOR agonist, on spontaneous synaptic transmission, and excitability of D1 and D2 dopamine receptor-expressing MSNs (Ma et al., 2012). Thus, while acute application of this opioid reduced the frequency of spontaneous excitatory and inhibitory postsynaptic currents in both regions, the effect was greater in the VMS, in particular in the nucleus accumbens (NAc) shell, where excitatory currents from D2 cells and inhibitory currents from D1 cells were decreased by the largest amount. DAMGO also increased cellular excitability in the VMS, as shown by reduced threshold for evoking action potentials (APs). These results indicated that the VMS is a critical mediator of DAMGO effects (Ma et al., 2012).

In a subsequent study, we examined the membrane and synaptic properties of MSNs in the NAc of mice trained to self-administer the opioid remifentanil. We found that prior opioid exposure did not alter the basic membrane properties nor the kinetics or amplitude of miniature excitatory postsynaptic currents (mEPSCs). However, when challenged with DAMGO, the characteristic inhibitory profile was reduced in D1- but not in D2-MSNs from mice receiving remifentanil, suggesting a D1 pathway-specific effect associated with the acquisition of opioid-seeking behaviors (James et al., 2013). While the NAc has received particular attention in addiction research, changes in the DLS could be implicated in the transition to habitual behavior after acquisition of drug-seeking (Yin et al., 2004; Malvaez and Wassum, 2018; Lipton et al., 2019). Thus, more studies on the role of the DLS, specifically designed to tease apart potentially differential effects of opioids on direct and indirect pathway MSNs are needed, as both pathways are implicated in the control of voluntary actions (Cui et al., 2013).

Until recently, most electrophysiological studies aimed to address this question have used mouse brain slices. However, these *in vitro* reduced preparations do not allow correlation of electrophysiological changes with addiction behaviors. In recent years, new imaging techniques have provided a rich armamentarium to study brain activity and behavior simultaneously. Ca^2+^ influx produced by action potential firing can be visualized with fluorescent Ca^2+^ indicators and is commonly used as a proxy of neuronal activity. Two-photon laser-scanning microscopy (2PLSM) has been traditionally the method of choice to examine Ca^2+^ transients evoked by AP. Usually, the head of the animal is fixed and the body rests on a styrofoam ball, which limits the rich behavioral repertoire and may alter neuronal activity (Donzis et al., 2020). Miniaturized microscopes (Miniscopes) are becoming a powerful tool to examine neuronal activity in more ethological, freely moving conditions (Ghosh et al., 2011; Stamatakis et al., 2021).

In the present study, we combined viral expression of a genetically encoded Cre-dependent Ca^2+^ indicator, GCaMP6f, with the use a Miniscope to image spontaneous Ca^2+^ activity in direct and indirect pathway MSNs in the DLS, or cerebral cortex of freely moving mice before and after systemic injection of oxycodone or saline as control. Following the *in vivo* studies, mouse brains were dissected and used for *in vitro* Ca^2+^ imaging or electrophysiological recordings. Convergent data demonstrated overall inhibitory effects of oxycodone on MSNs from both direct and indirect pathways. In contrast, cortical pyramidal neuron activity was increased.

## Materials and methods

### Mice and surgery

Mice aged 3–4 months were obtained from our breeding colonies at the University of California Los Angeles (UCLA). All experimental procedures were performed in accordance with the United States Public Health Service Guide for Care and Use of Laboratory Animals and were approved by the Institutional Animal Care and Use Committee at UCLA. Every effort was made to minimize pain, discomfort, and the number of mice used. Animal housing conditions were maintained under a standard 12 h light/dark cycle (light cycle starting at 6 AM and ending at 6 PM) and at a temperature of 20–26°C. The animals had *ad libitum* access to food and water.

To examine the effects of opioids on direct and indirect pathway MSNs, D1- (*n* = 3) and A2A-Cre (*n* = 3) mice of either sex were injected bilaterally at stereotaxic coordinates, relative to Bregma (in mm): +1 A/P, ±2 M/L, -3.3 D/V (dorsal striatum) with 1 μl of an adeno-associated virus (AAV) containing GCaMP6f. After 2 weeks, the cortical tissue overlying the striatum was aspirated to allow implantation of a GRIN lens (1.8 mm, Edmund Optics, Barrington, NJ, USA) with dental cement (Supplementary Figures 1B,C). 1–2 weeks later, a baseplate to hold the open-source UCLA Miniscope (V3) was affixed to the skull with dental cement. For comparison, to examine the effects of opioids on cortical pyramidal neurons (CPNs), a separate group of mice (3 month-old) were injected with 0.5 μl of an AAV containing GCaMP6f expressed using either the pan-neuronal Syn promoter (*n* = 2 mice) or the more selective for excitatory neurons CaMKII promoter (*n* = 2 mice) at stereotaxic coordinates, relative to Bregma (in mm): +0.5 A/P, +1 M/L, and -0.6 D/V, corresponding to the motor M1 region (Supplementary Figure 1A). After 2 weeks, a thin layer of cortical tissue was aspirated to allow implantation of the GRIN lens (1.8 mm, see above). 1–2 weeks later a baseplate to hold the Miniscope was affixed to the skull.

### Behavioral assessments and Miniscope imaging

Mice were placed in a cylindrical behavioral chamber (30 cm in diameter and 30 cm high) after a Miniscope was attached to the baseplate. They were allowed to acclimate to wearing the Miniscope and moving freely in the chamber (1 hr for three consecutive days). Both Ca^2+^ transients acquired with a V3 Miniscope and behavioral (webcam) data were collected using the Data Acquisition Software (DAQ, UCLA, Los Angeles, CA, USA) simultaneously for ∼5 min. Ca^2+^ transients were captured at 20 Hz with maximum exposure and gain. Behavioral videos were captured between 20 and 22 Hz due to the webcam frame rate. After baseline data were acquired, mice were injected with either oxycodone (3 mg/kg, IP) or saline as control. The final volume ranged between 0.2 and 0.3 ml. Recordings were taken at 5 and 15 min after oxycodone or saline administration, but mostly data from 15 min are reported. As we were only interested in the acute effects of systemic administration of oxycodone, each mouse was used only 1–2 times to avoid opioid receptor sensitization.

### Behavioral and Ca^2+^ transient analyses

The number of mice, videos, and cells used for behavioral and Miniscopes data analyses are shown in Supplementary Table 1. Behavioral data were processed using the Python-based ezTrack video analysis pipeline (Pennington et al., 2019). Using the location tracing module, an animal’s center of mass (COM) was tracked across each session. A reference frame was defined, with each frame compared to the reference frame to locate the animal’s COM. The COM was saved, and the module created both traces and a heat map of the final output. Distance traveled was calculated in the software by measuring a specific distance in the module (in pixels) and converting this pixel distance to actual length (cm) based on the dimensions of the behavioral chamber. Animal movement was also analyzed manually through visual examination of each frame of the behavioral videos, receiving a score of 0 (non-movement) and 1 (movement). Lastly, the percentage of movement was calculated by dividing the number of movement frames by the total number of frames for each behavioral video.

Ca^2+^ transient activity from Miniscope video recordings was processed using MiniAn, an open-source Miniscope analysis pipeline (Dong et al., 2022). This pipeline performs background subtraction, rigid motion correction using the NormCorre algorithm, and source extraction using the constrained non-negative matrix factorization (CNMF) algorithm. MiniAn produces the estimated footprint, Ca^2+^ activity trace and the deconvolved activity trace of each neuron. To examine the same neurons across different recording sessions before and 15 min after drug administration we used the Python-based program Cross-reg, which is part of the MiniAn package. This software aligned the spatial footprints of the neurons and considered the neuron to be the same across the recordings if the footprints were within a five pixel threshold of movement between sessions. The Cross-reg program then outputs the neurons that are matched between sessions, and across all sessions in total. A threshold was manually set for each Ca^2+^ trace to identify peaks. The minimum threshold was set to the RMS of the trace and increased if the threshold did not appropriately remove noise. Neurons with non-physiological Ca^2+^ traces, e.g., continuous shifts of the baseline, also were manually removed. In addition, since after viral injection not only cell bodies but also the neuropil show fluorescence, we set up several criteria to select only what the program and our best estimates indicated high probability of detecting neuronal somata. These included a circular or ovoid shape, an area ranging from 200 to 400 μm^2^ (average ∼294 ± 23 μm^2^ from a random sample of *n* = 10 cells), and only objects exhibiting changes in fluorescence (Donzis et al., 2020). Thus, in contrast to fiber photometry, which detects mainly changes in the neuropil (Legaria et al., 2022), the Miniscopes allowed for more selective measurements of neuronal activity after elimination of spurious objects.

### Two-photon laser-scanning microscopy and electrophysiology in slices

After the last Miniscope imaging recording, some mice were sacrificed and brain slices containing cerebral cortex and striatum (350 μm) were obtained for electrophysiological recordings and Ca^2+^ imaging *in vitro* using 2PLSM. Ca^2+^ imaging was performed using a Scientifica 2PLSM equipped with MaiTai laser (Spectra-physics). To image Ca^2+^ signals, the laser was tuned at 920 nm wavelength. The laser power measured at the sample was less than 30 mW with a 40 × water-immersion objective lens (Olympus, Tokyo, Japan). Ca^2+^ transients were sampled at a rate of 3.81 Hz using SciScan software (Scientifica, Uckfield, UK). Optical signal amplitudes were expressed as Δ*F*/*F*, where *F* represents the resting light intensity at the beginning of the optical trace, and Δ*F* represents the change in peak fluorescence during the biological signal. These values were then multiplied by 100 to obtain percentage values. ImageJ (NIH) was utilized to process raw fluorescence values, which were then exported to Excel (Microsoft, Redmond, WA, USA) and Clampfit software to calculate % Δ*F*/*F*.

### Electrophysiological recordings

The effects of oxycodone on membrane and synaptic properties were examined in labeled and unlabeled MSNs. Only the intact (non-aspirated) side was used. In a separate group of control mice not used for Miniscope recordings, whole-cell patch clamp recordings were obtained from unidentified MSNs. Coronal slices containing cerebral cortex and striatum were perfused with oxygenated ACSF and maintained in the chamber at room temperature. Whole-cell patch clamp recordings from MSNs were obtained in voltage clamp mode using Cs-methanesulfonate as the pipette solution, or in current clamp mode using K-gluconate as internal solution (Holley et al., 2022). After determination of basic membrane properties, including cell membrane capacitance, input resistance, and decay time constant, spontaneous synaptic activity was recorded. At -70 mV holding potential, most synaptic events are glutamatergic (excitatory post-synaptic currents, sEPSCs) and mediated by α-amino-3-hydroxy-5-methyl-4-isoxazolepropionic acid (AMPA) receptors. At a holding potential of +10 mV, most events are GABAergic (inhibitory post-synaptic currents, sIPSCs) and mediated by GABA_*A*_ receptors. At this holding potential (+10 mV), after 3–5 min recording in control conditions, oxycodone (1 μM) was added to the external ACSF solution. The recording at this potential continued for at least 8 min and then the potential was reverted to -70 mV to examine the effects of oxycodone on glutamatergic activity. Measures of basic membrane properties used the Clampfit program and the frequency of spontaneous synaptic activity was calculated with the miniAnalysis program.

### Statistics

There were three levels of analysis in our study, mice, video recording sessions, and cells. Most animals were tested with oxycodone only once to prevent opioid receptor sensitization. One A2A mouse and one Syn mouse were tested twice to determine reproducibility of the response to the drug. The effects of oxycodone were assessed by comparing number of cells and their frequencies per video (session) analyzed. Values are expressed as mean ± SEM. Statistical significance was calculated using SigmaPlot (v. 14). Data were analyzed by Student’s *t*-tests, paired or unpaired, as well as one- and two-way ANOVAs with or without repeated measures (RM). Pairwise multiple comparison procedures used appropriate *post-hoc* tests. Differences between or among groups were considered significant if *p* < 0.05.

## Results

### Mouse behavior before and after acute systemic injection of oxycodone or saline

A single dose of oxycodone (3 mg/kg, IP) was selected for these studies. This is a relatively high dose more likely to replicate both analgesic and addictive properties of oxycodone in mice. In a previous study, we found that this dose induces hyperkinetic behavior and, when repeated for three consecutive days, it also induces locomotor sensitization, which does not occur in mice lacking MORs in striatal neurons from the direct pathway (D1 receptor-expressing) (Severino et al., 2020). Another study found that doses between 2 and 4 mg/kg (subcutaneous) produce maximal analgesia starting at ∼3 min after systemic injection (Nasseef et al., 2019).

**FIGURE 1:**
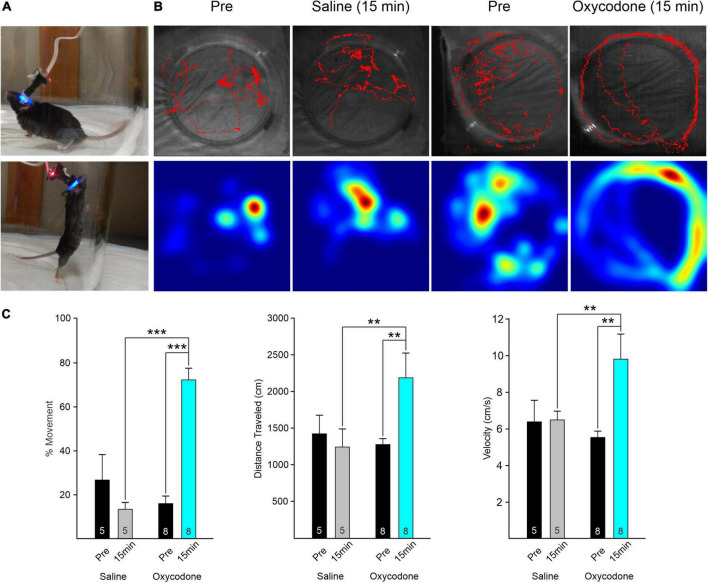
**(A)** Mouse behavior in observation chamber with Miniscope attached. The mouse could move and rear freely. **(B)** Movement of the mouse was tracked using a webcam placed above the arena. Red traces (upper panels) track the behavior of the mice during the ∼5 min recording. Heat maps (lower panels) indicate the place where the mouse spent most of the time. Movement analysis was performed with ImageJ and Manual Tracking plugin. Behavior of an A2A-Cre mouse before (Pre) and 15 min after injection of saline. The movement patterns were similar. Traces of the same A2A-Cre mouse before (Pre) and 15 min after injection of oxycodone (3 mg/kg). Mouse continuously circled the outer perimeter of the arena after oxycodone. In addition, we observed a Straub tail ∼3 min after injection and absence of rearing. **(C)** Graphs indicate the % of frames that the mouse was moving (left graphs) before and 15 min after injection of saline or oxycodone. In saline, there was a statistically non-significant reduction in movement. After oxycodone, movement increased significantly. Middle graphs indicate total distance traveled during the recording session. The distance increased significantly after oxycodone and the velocity (right graphs) increased likewise. Numbers within the bar graph indicate the number of videos examined. Data were analyzed by two-way RM ANOVA with Holm–Sidak *post-hoc t*-test, **p < 0.01, ***p < 0.001.

One week following baseplate implantation the mice were allowed to acclimate to the recording chamber and to the Miniscope. They quickly adapted to both and the weight of the Miniscope did not perturb the ability of the mice to move and explore freely (Figure 1A). Before oxycodone administration, mice explored the behavioral arena, reared, and groomed normally. After oxycodone administration, typical signs of MOR activation occurred. These included the typical Straub tail starting ∼3 min after injection, increased locomotion after 5–6 min, and circling behavior (Figure 1B). In addition, the mice moved swiftly in a tiptoeing manner. Rearing episodes occurred at least once before oxycodone, whereas they were completely absent after oxycodone.

To determine the time course of oxycodone effects, as a first approximation, we took videos before (Pre), and at 5 and 15 min after systemic injection of oxycodone. We noticed that, while behavioral changes (e.g., hyperkinesia), were already apparent 5 min after oxycodone, changes in Ca^2+^ activity were more progressive and effects were more pronounced at 15 min compared with 5 min after oxycodone (Supplementary Figure 2). Thus, for subsequent statistical analyses we compared only Pre and 15 min after oxycodone or saline. This time coincides with maximal effects of oxycodone, based on pharmacokinetics in rodents (Huang et al., 2005; Nasseef et al., 2019).

We first measured the total amount of time the mouse displayed in movement *versus* non-movement before and 15 min after saline or oxycodone, regardless of Cre expression (D1- or A2A-Cre) or brain region injected with the Ca^2+^ reporter (striatum or cortex) (*n* = 5 sessions for saline and *n* = 8 for oxycodone). For that purpose, we determined the number of frames where movement was detected and divided by the total number of frames to obtain a percentage of movement. The duration of each frame was 45–50 ms based on webcam framerate. There was a statistically significant interaction between treatment and time (two-way ANOVA, *F*_1,22_ = 31.9, *p* < 0.001). The percent of time moving increased significantly after oxycodone administration (Pre *versus* 15 min, *p* < 0.001, Holm–Sidak *post-hoc t*-test), whereas after saline, if anything, there was a non-significant decrease in movement (Pre *versus* 15 min, *p* = 0.08, Holm–Sidak *post-hoc t*-test) (Figure 1C). The total distance mice traveled before and 15 min after oxycodone administration also was determined. Similar to percent movement, there was a statistically significant interaction between treatment and time (two-way ANOVA, *F*_1,22_ = 4.37, *p* = 0.048). The distance traveled increased significantly after oxycodone administration (Pre *versus* 15 min, *p* = 0.01, Holm–Sidak *post-hoc t*-test), whereas after saline there was practically no change in distance traveled (Pre *versus* 15 min, *p* = 0.66, Holm–Sidak *post-hoc t*-test). As for velocity, the difference in the mean values between Pre and post injection conditions was statistically significant when the different treatments were taken into consideration (two-way ANOVA, *F*_1,22_ = 4.5, *p* = 0.045). Thus, the velocity significantly increased after oxycodone (Pre *versus* 15 min, *p* = 0.003, Holm–Sidak *post-hoc t*-test), whereas it remained constant 15 min after saline injection (Pre *versus* 15 min, *p* = 0.95, Holm–Sidak *post-hoc t*-test). Effects began to subside 30 min after oxycodone injection (not shown).

### Oxycodone reduces the number of fluorescent medium-sized spiny neurons from direct and indirect pathways

**FIGURE 2:**
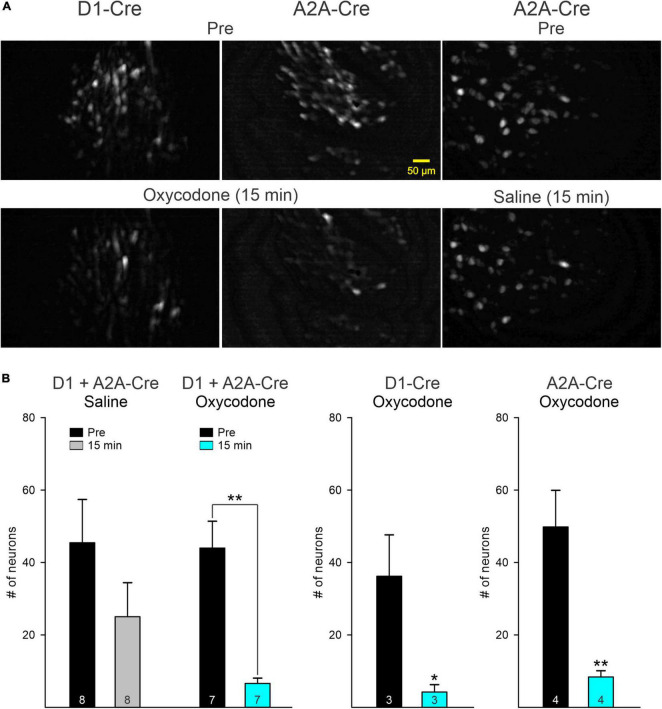
**(A)** Maximum intensity projection panels illustrate fluorescent medium-sized spiny neurons (MSNs) visualized with the aid of a Miniscope before (Pre) and 15 min after IP injection of oxycodone or saline. Notice the overall reduction in fluorescence after oxycodone in D1- and A2A-Cre mice. In contrast, fluorescence intensity was only slightly reduced after saline injection. **(B)** Graphs indicate that the main driver of reduced fluorescence intensity was a significant reduction in the number of MSNs detected by the Miniscope in both D1- and A2A-Cre mice. Saline injection induced a non-significant reduction in the number of neurons, likely reflecting reduced movement as shown in Figure 1C. Graphs on the left show the comparison between saline and oxycodone treatment after grouping together D1 and A2A MSNs (data were analyzed by the two-way ANOVA). Graphs on the right show the effects of oxycodone with D1 and A2A MSNs separated. After oxycodone the reduction in the average number of fluorescent MSNs was statistically significant (for D1 MSNs, *p* = 0.049 and for A2A MSNs, *p* = 0.0066, Student’s *t*-test). *p < 0.05, **p < 0.01. Numbers inside bars indicate number of recording sessions.

After oxycodone injection, the overall fluorescence in the field of view was reduced significantly. The major contributor of this reduction was a decrease in the number of visible cells. In contrast, after saline injection only a small, non-significant decrease was detected (Figures 2A,B) and appeared to be correlated with less overall movement (see Figure 1C). When grouping together D1 and A2A MSNs, there was a statistically significant difference in the mean values between Pre and post injection conditions when the saline *versus* oxycodone treatments were taken into consideration (two-way ANOVA, *F*_1,26_ = 10.6, *p* = 0.003). After oxycodone there was a statistically significant reduction in the number of visible neurons (*p* = 0.008, Holm–Sidak *post-hoc t*-test), whereas after saline there was no change (*p* = 0.1, Holm–Sidak *post-hoc t*-test).

### Contrasting changes in Ca^2+^ transient frequency in direct and indirect pathway medium-sized spiny neurons after oxycodone

**FIGURE 3:**
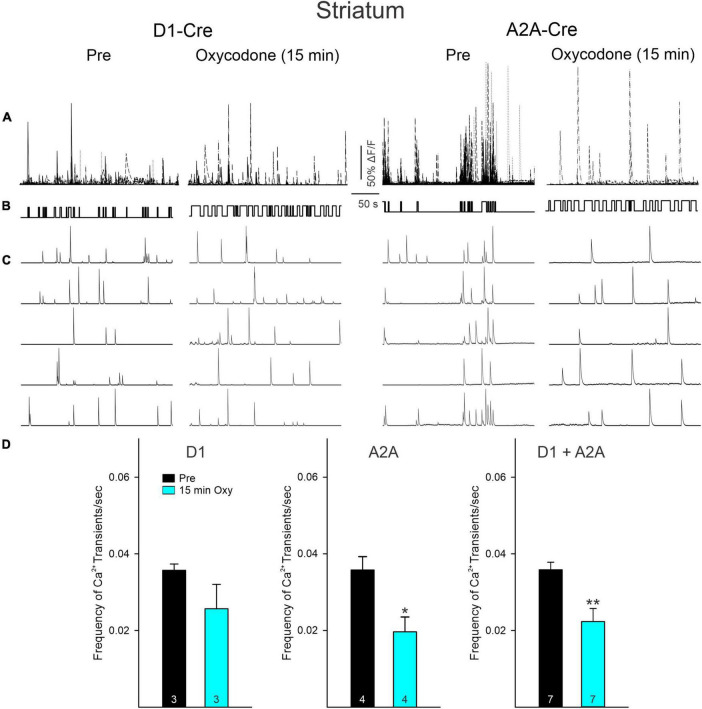
**(A)** Traces represent multi-neuronal Ca^2+^ transients recorded in D1- and A2A-Cre mice before (Pre) and 15 min after oxycodone. Each trace represents the activity of 1 medium-sized spiny neuron (MSN). Notice that the number of active cells is reduced after oxycodone. **(B)** Upward deflections indicate the mouse was moving. In control conditions the behavior appeared more random, whereas after oxycodone it became more stereotyped. **(C)** Some representative Ca^2+^ transient traces of single MSNs. Notice the tight correlation between Ca^2+^ transients and behavior, more noticeable in A2A Pre. After oxycodone injection, this correlation was lost, suggesting decoupling between striatal neuron activity and movement. **(D)** Graphs illustrate changes in frequency of Ca^2+^ transients before and after injection of oxycodone or saline in striatal MSNs. When analyzed separately, D1 MSNs only displayed a discrete, non-significant reduction in frequency. In contrast, the frequency in A2A MSNs was significantly reduced. When grouped together, cells from D1- and A2A-Cre mice displayed a significant decrease in frequency after oxycodone. *p < 0.05, **p < 0.01, Student’s *t*-test.

Ca^2+^ transient frequency of striatal MSNs (D1 + A2A) remained unchanged before and after saline injections (Supplementary Figure 4). Further, before oxycodone and before or 15 min after saline injection, multi-neuronal Ca^2+^ activity was tightly associated with movement (Figures 3A–C and Supplementary Figures 3A, 4). To determine this association, we calculated the frequency of Ca^2+^ transients during epochs of movement *versus* non-movement. Movement was defined as a clear and measurable displacement of the body within the arena. Small head movements were not included in the movement category. Regardless of treatment, prior to injection the mice spent most of the time in non-movement compared with movement (79 ± 5% *versus* 21 ± 5%). During episodes of immobility, the frequency of Ca^2+^ transients was significantly reduced compared with episodes of movement (Supplementary Figure 3A). We then compared the effects of oxycodone on the frequency of Ca^2+^ transients Pre and 15 min after injection. There was an overall reduction in frequency after oxycodone (D1 + A2A, *p* = 0.0048, Student’s *t*-test). However, while the reduction in frequency observed in D1-MSNs was not statistically significant (*p* = 0.20, Student’s *t*-test), the reduction in A2A-MSNs Ca^2+^ transient frequency was statistically significant (*p* = 0.021, Student’s *t*-test) (Figure 3D). This suggests that the overall reduction in frequency of striatal neurons after oxycodone is driven primarily by MSNs constituting the A2A adenosine receptor-tagged indirect pathway. In agreement, even though the frequency of Ca^2+^ transients was not significantly reduced in D1-MSNs after oxycodone, their activity still maintained a positive correlation between number of Ca^2+^ transients during movement *versus* non-movement, this correlation was lost in A2A-MSNs (Supplementary Figure 3A). We also noticed that after oxycodone there was a dissociation between Ca^2+^ transient activity and behavior, which became more stereotyped compared to the more random behavior in Pre oxycodone conditions (see Figure 3B).

### Effects of oxycodone on Ca^2+^ transient activity in cortical pyramidal neurons

**FIGURE 4:**
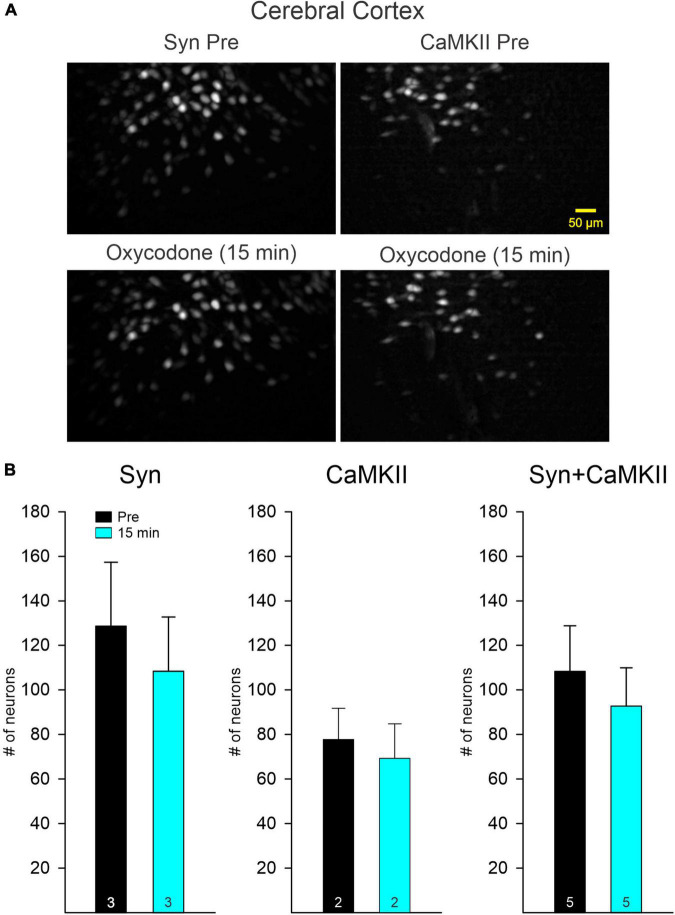
**(A)** Maximum intensity projection panels illustrate fluorescent cortical pyramidal neurons (CPNs), Syn (left), and CaMKII (right), visualized with the aid of a Miniscope in Pre conditions and 15 min after IP injection of oxycodone. The number of CPNs did not seem to change after oxycodone IP injections. **(B)** Graphs show the quantification of neurons detected before and 15 min after injection of oxycodone. No significant changes were observed in the total number of neurons in both Syn and CaMKII mice when examined separately or when pooled together.

**FIGURE 5:**
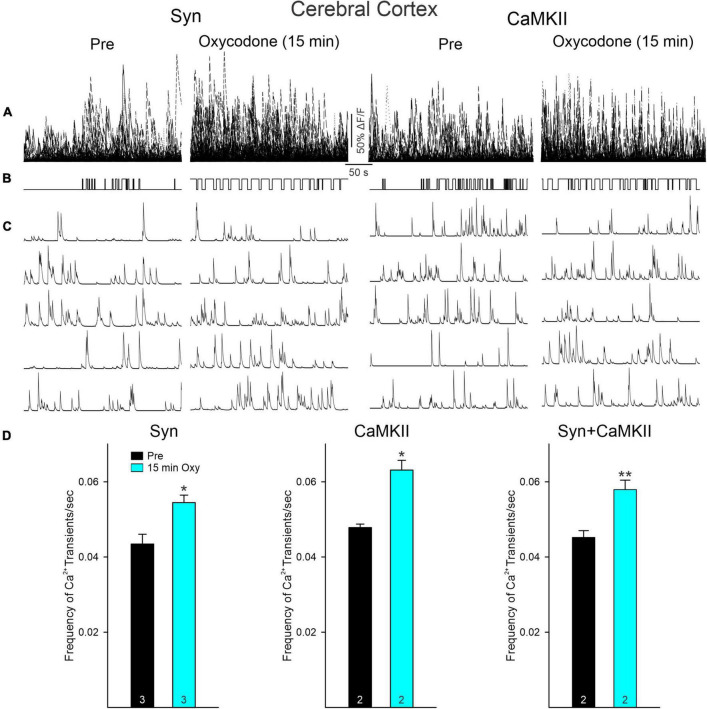
**(A)** Traces represent multi-neuronal Ca^2+^ transients recorded in the cortex (layers II/III) of mice with viral injections of GCaMP6f expressed using Syn or CaMKII promoters before and 15 min after oxycodone. Each trace represents the activity of 1 neuron. Notice that, in contrast to striatum, the number of active cells was not reduced after oxycodone. **(B)** Upward deflections indicate the mouse was moving. As shown in the previous figure, in control conditions the behavior was random, whereas after oxycodone it became more stereotyped. **(C)** Representative Ca^2+^ transient traces of single cortical pyramidal neurons (CPNs). After oxycodone, Ca^2+^ transient frequency increased. **(D)** Graphs show changes in frequency of Ca^2+^ transients before and after injection of oxycodone in CPNs. Cells from Syn and CaMKII mice increased their frequency after oxycodone when analyzed separately or when pooled together. *p < 0.05, **p < 0.01.

To determine if opioid administration causes generalized depression of neuronal activity and as a comparison with striatal changes, we also examined the effects of oxycodone on CPNs. Mice were injected with the Ca^2+^ indicator using a Syn (*n* = 2) or a CaMKII (*n* = 2) promoter to more selectively infect excitatory CPNs. The number of CPNs infected with the Ca^2+^ indicator was consistently higher than that in striatal MSNs. The overall frequency of Ca^2+^ transients also was consistently higher than that of MSNs. Similarly, the number of CPNs infected with the Syn promoter was higher than that of CPNs infected with the CaMKII promoter (Figure 4). As changes induced by oxycodone in CPNs infected with either promoter were similar, and considering that CPNs are the most abundant cell types in the cerebral cortex, data were analyzed separately or pooled together. Notably, the number of CPNs visualized Pre and 15 min after oxycodone injection did not change significantly when all CPNs (Syn + CaMKII) were considered (*p* = 0.577, Student’s *t*-test) or when separated (Syn *p* = 0.618, CaMKII *p* = 0.723, Student’s *t*-test), indicating that the depression of Ca^2+^ activity observed in striatum did not occur in the cortex (Figures 4A,B). More remarkably, the frequency of Ca^2+^ transients was significantly increased after oxycodone (all CPNs combined *p* = 0.003, Syn CPNs *p* = 0.028, and CaMKII CPNs *p* = 0.029, Student’s *t*-test) (Figure 5), suggesting that DLS is an unlikely substrate of behavioral hyperactivity after oxycodone. Interestingly, the correlation between Ca^2+^ transient activity and behavior was less evident in cortex compared to stratum. Thus, while the Ca^2+^ transient frequency was slightly reduced during non-movement compared to movement, this correlation was lost after oxycodone, during which the number of Ca^2+^ transients was practically equal during movement and non-movement (Supplementary Figure 3B). Some examples of individual MSNs and CPNs before and after oxycodone can be seen in Supplementary Figure 5. In addition, raw videos (without motion correction or background subtraction) illustrating effects of oxycodone in striatum and cortex are available in the Supplementary Videos 1–6.

### Slice electrophysiology confirmed Ca^2+^ imaging findings in medium-sized spiny neurons

**FIGURE 6:**
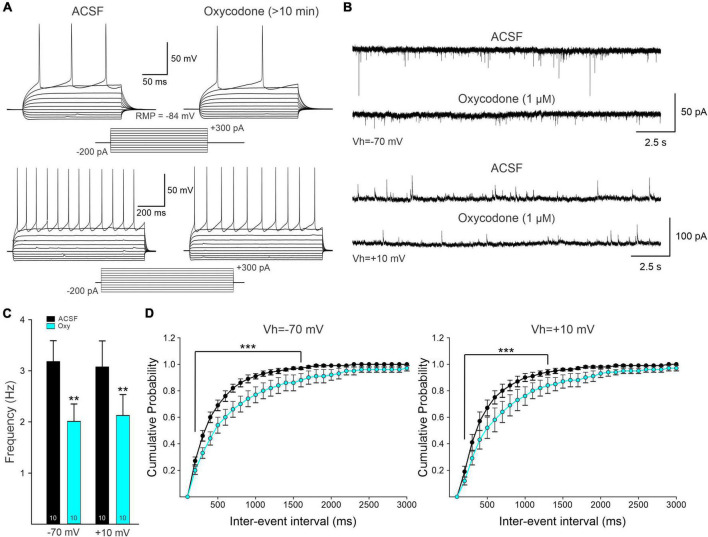
**(A)** Representative electrophysiological recordings of a D1-Cre medium-sized spiny neuron (MSN) in current-clamp mode. Current injections of +300 pA (250 ms duration, upper traces; 1 s duration, lower traces) generated a train of action potentials (APs). Bath application of oxycodone decreased the frequency of evoked APs. **(B)** Whole-cell patch clamp recordings from intact mice not used for Miniscope recordings. Spontaneous postsynaptic currents of MSNs held at -70 and +10 mV to electrophysiologically isolate putative excitatory [α-amino-3-hydroxy-5-methyl-4-isoxazolepropionic acid (AMPA) receptor-mediated] and inhibitory (GABA_A_ receptor-mediated) synaptic currents. Ten min after oxycodone bath application, spontaneous synaptic activity was reduced. This reduction is likely the result of presynaptic inhibition of neurotransmitter release. **(C)** Average frequency of spontaneous synaptic currents recorded at -70 mV and +10 mV. Numbers within the bar graphs indicate the number of neurons recorded. In both conditions, there was a statistically significant reduction in frequency (Student’s *t*-test). **(D)** Average cumulative probability of inter-event intervals of spontaneous postsynaptic currents before and after oxycodone. A significant reduction in frequency occurred after oxycodone (two-way RM ANOVA, Bonferroni *post-hoc* test). **p < 0.01, ***p < 0.001.

After completing the Miniscope recordings, some mice were sacrificed, the baseplate and lens removed, and the brains extracted and sliced to determine the location and extent of the viral injection. Visual and microscopic examination confirmed the location above the dorsal striatum, with no damage extending into this region. However, as in these mice the GCaMP6 viral injections were bilateral, only the intact side was used for slice electrophysiology (Supplementary Figure 1D). Most of the recordings were from fluorescent-positive cells (*n* = 7 from 6 different mice). In the case of D1- or A2A-Cre, if a cell was not fluorescent, it was considered as belonging to the other pathway, i.e., a negative cell from a D1-Cre animal was assumed to belong to the indirect pathway and *vice versa*. Data from Cre animals were pooled. In current clamp mode, oxycodone administration in brain slices caused a small membrane hyperpolarization (2–3 mV) in all but 1 MSN. This hyperpolarization was enough to cause an increase in inward rectification and reduction in membrane input resistance. As a consequence, cell excitability decreased in all but 1 cell (7 ± 2 APs in control conditions and 5.2 ± 1.6 after oxycodone, with 1 s duration pulses) (Figure 6A). The difference however, did not reach statistical significance (*p* = 0.17, paired *t*-test). Finally, similar to the previous voltage clamp recordings in intact animals, the spontaneous synaptic activity recorded at resting membrane potential was reduced significantly 10 min after oxycodone application (3.46 ± 0.4 Hz in control conditions and 2.48 ± 0.2 Hz after oxycodone, *p* = 0.024, paired *t*-test).

Electrophysiological data on the effects of bath application of oxycodone (1 μM) also were collected from intact WT mice (*n* = 5) not used for Miniscope imaging. Under infrared videomicroscopy, cells had the typical size, and shape of striatal MSNs (*n* = 10). In addition, basic membrane properties measured in voltage clamp also corresponded to those of MSNs and had an average cell membrane capacitance of 136 ± 13 pF, input resistance of 47.6 ± 4.3 MΩ, and a decay time constant of 2.3 ± 0.2 ms. The spontaneous synaptic currents recorded at -70 mV (putative EPSCs) were reduced after acute application of oxycodone (3.2 ± 0.4 Hz control and 2.0 ± 0.3 Hz after oxycodone, *p* = 0.004, paired *t*-test) (Figures 6B–D). At +10 mV, the average frequency of spontaneous synaptic currents (putative IPSCs) also was significantly reduced by acute application of oxycodone (3.1 ± 0.4 Hz in control conditions and 2.1 ± 03 Hz after the drug, *p* = 0.008, paired *t*-test). Overall, the electrophysiological data confirmed a generalized reduction of MSN excitability and synaptic inputs in DLS.

### Effects of oxycodone on Ca^2+^ activity visualized with two-photon laser-scanning microscopy in striatal slices

**FIGURE 7:**
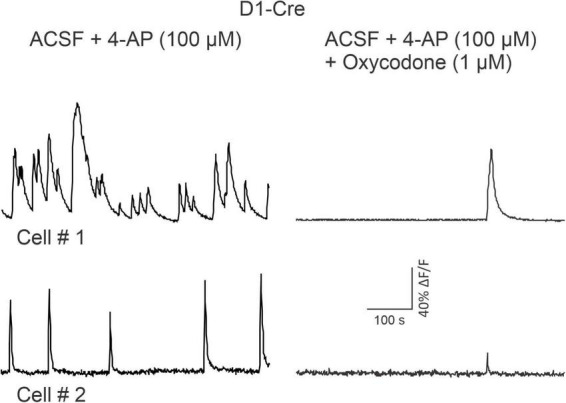
Traces are from two medium-sized spiny neurons (MSNs) recorded *in vitro* after a D1-Cre mouse used for Miniscope recordings was sacrificed. Two-photon laser-scanning microscopy (2PLSM) was used to identify MSNs expressing the Ca^2+^ reporter GCaMP6f. MSN activity was promoted by bath application of 4-AP (100 μM) and is indicated by spontaneous, rhythmic Ca^2+^ transients. 10 min after oxycodone, spontaneous activity was severely reduced. See Supplementary Videos 7–8.

As oxycodone increased locomotor activity, we expected to observe an increase in Ca^2+^ transient activity in D1 MSNs. In order to confirm results from Miniscope imaging, some slices from D1-Cre mice also were used for 2PLSM imaging after *in vivo* recordings were completed. Because striatal MSNs *in vitro* do not fire APs spontaneously due to highly hyperpolarized resting membrane potentials, cells were activated with the K^+^ channel blocker 4-aminopyridine (4-AP, 100 μM). By blocking A-type K^+^ currents, 4-AP increases neurotransmitter release and induces spontaneous membrane oscillations. Alternatively, APs were evoked by intracellular injection of depolarizing currents. In all cases, oxycodone caused a reduction in both Ca^2+^ transient frequency (from 5.75 events to just 1/600 s, *p* = 0.05, paired *t*-test) and amplitude (52% reduction, *p* = 0.008, paired *t*-test) of MSNs in D1-Cre mice (*n* = 7 cells, in 6 slices from 3 D1-Cre mice) (Figure 7 and Supplementary Videos 7, 8).

## Discussion

The Miniscope technology applied to freely behaving animals is fast becoming the method of choice to correlate neuronal activity and behavior. It is also helping to elucidate the neurobiological mechanisms of addiction (Vickstrom et al., 2021). Ca^2+^ transients are a bona fide representation of neuronal firing, albeit with inherent limitations due to the slow kinetics of fluorescent Ca^2+^ reporters. Notwithstanding this limitation, the wealth of information provided by Miniscope Ca^2+^ imaging is substantial. In combination with available Cre-expressing mice, this technology also allows examination of neuronal activity of discrete cell populations. In the present study, we used D1- and A2A-Cre mice to express the fluorescent Ca^2+^ indicator in MSNs of either the direct or indirect striatal output pathways, respectively. At a dose of 3 mg/kg, oxycodone increased locomotor activity. This is in line with our previous (Severino et al., 2020) and other studies (Liu et al., 2005). Given the increase in locomotor activity following oxycodone, our initial hypothesis was that cells of the direct pathway would be more active after oxycodone administration, whereas cells of the indirect pathway would be unaffected or perhaps inhibited. To our surprise, MSNs in both pathways demonstrated attenuated activity as manifested by significantly reduced numbers of detectable cells after oxycodone. The frequency of Ca^2+^ transients was significantly reduced in indirect pathway MSNs, whereas the reduction in direct pathway MSNs was more subtle and did not reach statistical significance. In addition, while the frequency of Ca^2+^ transients was higher during movement *versus* non-movement before oxycodone, this correlation was lost in indirect pathway MSNs but not in direct pathway MSNs after opioid administration. Opposite effects of oxycodone on behavior and Ca^2+^ transient activity suggest a dissociation between striatal activity and behavior. Interestingly, a pharmacological MRI study demonstrated that even a single dose of oxycodone (2 mg/kg, IP) results in generalized reduction of functional connectivity in mice (Nasseef et al., 2019). Our results also suggest that other brain areas, including the cerebral cortex, are associated with increased locomotor activity after oxycodone. In particular, recent studies indicate that CPNs encode information related to the onset and offset of motor sequences and this information is relayed to different striatal circuits (Nelson et al., 2021). In the case of opioid administration, it is likely that this information transfer is blunted by presynaptic inhibition of glutamate release by MORs.

Our electrophysiological and 2PLSM studies in slices corroborated primarily inhibitory effects of oxycodone on MSNs. Activity-dependent Ca^2+^ transients induced by depolarizing current pulses or 4-AP, were significantly reduced by oxycodone bath application. In addition, electrophysiological recordings in current clamp mode demonstrated that after drug application the membrane potential became more hyperpolarized, inward rectification increased, and the number of APs decreased. This is consistent with our previous studies showing that DAMGO, a selective MOR agonist induces a rightward shift in the current-response curve of D1 and D2 (GFP-labeled) MSNs in the DLS (Ma et al., 2012) and could explain the reduced number of visible neurons after oxycodone administration. The effects of oxycodone on sEPSCs, i.e., reduced event frequency, are consistent with presynaptic regulation of neurotransmitter release by MOR activation. Indeed, regulation of glutamatergic transmission by presynaptic MORs has been conclusively demonstrated (Atwood et al., 2014b). For example, DAMGO induces long-lasting reduction of electrically evoked EPSC amplitude in MSNs of the DLS and a single *in vivo* exposure to oxycodone can disrupt MOR-induced long-term depression (LTD) (Atwood et al., 2014a). Further, MORs specifically located on thalamostriatal terminals (VGluT2-expressing) can inhibit glutamate release and, behaviorally, MORflox-VGluT2cre mice do not acquire conditioned place preference and fail to display oxycodone-induced locomotor stimulation (Reeves et al., 2021). However, VGlut2-expressing neurons also are present in other regions including, among others, amygdala, hypothalamus, cerebellum, and cerebral cortex, implying that other pathways also could be involved.

Single-cell extracellular and intracellular electrophysiological recordings *in vivo* have demonstrated that, in the absence of movement, a great percentage of striatal MSNs remain silent and, if spontaneously active, they discharge at low rates of ∼3–5 Hz (Sandstrom and Rebec, 2003; Mahon et al., 2006). Similarly, Ca^2+^ transient frequency is very low in the absence of movement and even during movement, this frequency remains low, probably due to the slow kinetics of GCaMP6. In agreement with other studies, the observed mean frequency in both D1- and A2A-Cre-labeled cells was less than 0.15 Hz (Klaus et al., 2017; Yu et al., 2018). As the firing rate of MSNs recorded electrophysiologically is considerably higher, it is likely that Ca^2+^ transients do not reflect single APs but burst firing, which is the result of convergent excitatory inputs from cortical and thalamic afferents. Presynaptic inhibition of glutamate release by MORs may prevent convergent inputs from generating burst firing and, in consequence, less frequent Ca^2+^ transients.

Before oxycodone, Ca^2+^ activity was positively correlated with movement. In contrast, after opioid administration, and in spite of increased movement, the number of active cells decreased and the frequency of Ca^2+^ transients also was significantly reduced, at least in A2A MSNs, suggesting a decorrelation between MSN activity and movement. This result is reminiscent of previous findings showing reduced population Ca^2+^ activity in MSNs from both direct and indirect pathways after cocaine IP injections, in spite of increased locomotion (Barbera et al., 2016). Interestingly, CPN numbers in our study did not change significantly after oxycodone and the Ca^2+^ transient frequency increased significantly. This suggests that cortical neurons play an important role in behavioral hyperactivity.

Our study has a number of important limitations. One is that MORs are known to be enriched in the patch *versus* the matrix compartment in DLS. Since GCaMP6 is present in any neurons that express Cre recombinase, future studies are needed to target GCaMP6 in the patch compartment. Also, oxycodone can bind, albeit with much lower affinity, to other types of opioid receptors. However, in preliminary experiments we also tested the effects of fentanyl, a more selective MOR agonist, and observed similar reductions in the number of visible neurons and Ca^2+^ transient frequency, suggesting that the main effects of oxycodone are mediated by MORs. We also need to emphasize that our study only examined acute effects of oxycodone in naïve mice and the outcomes might be different in the case of repeated injections of the drug (Iriah et al., 2019). In addition, the effects of oxycodone in MSNs of the NAc need to be examined as this region appears more sensitive to MOR activation than DLS (Ma et al., 2012). Finally, we need to consider that when imaging deep brain regions, the damage produced by aspiration of overlying tissue as well as by the lens itself can be extensive, even when using thin GRIN lenses.

## Conclusion

Our combined imaging and electrophysiological study demonstrates a dissociation between motor behavior and striatal direct and indirect pathway MSN activity after systemic administration of oxycodone. Instead of an expected increase in Ca^2+^ transient activity associated with increased locomotion, a reduced number of cells displayed Ca^2+^ transient activity. The frequency of these transients also was reduced, primarily in indirect pathway MSNs, probably due to membrane hyperpolarization and reduced burst firing. In contrast, the frequency of Ca^2+^ transients in projection neurons of the cerebral cortex increased significantly, suggesting an important role in behavioral activation. This implies that MOR activation hampers the communication along corticostriatal and thalamostriatal pathways, the main excitatory inputs onto MSNs, probably *via* presynaptic inhibition of glutamate release.

## Data availability statement

The raw data supporting the conclusions of this article will be made available by the authors, without undue reservation.

## Ethics statement

The animal study was reviewed and approved by the Institutional Animal Care and Use Committee at UCLA.

## Author contributions

JB, CC, ML, CE, and PG contributed to conception and design of the study. JB, KO, CY, AP, and DY organized the database. JB and CC performed the statistical analysis, wrote the first draft, and sections of the manuscript. All authors contributed to manuscript revision, read, and approved the submitted version.

## Supplementary material

Supplementary Figure 1 Example slices (350 μm) obtained from mice used for Miniscope recordings. **(A)** Cortical slice showing the site and spread of viral injection (CaMKII promoter) under infrared DIC optics (left panel) and under fluorescence microscopy (right panel). The injection corresponds to M1 motor cortex. **(B)** Example slice obtained from a D1-Cre mouse used for striatal Miniscope recordings. Left panel shows the extent of cortical tissue aspirated for GRIN lens implant. The spread of the viral injection remained circumscribed to the striatum, although it was common to see fluorescence along the corpus callosum and in the tissue surrounding the GRIN lens. **(C)** Higher magnification of a striatal slice from a D1-Cre mouse used for Miniscope recordings, but on the intact (non-aspirated) side. This slice was used for electrophysiological recordings of identified medium-sized spiny neurons (MSNs). The cell indicated by the yellow arrow was patched and the recording is shown in Figure 6A.

Supplementary Figure 2 To determine the time course of oxycodone effects we examined behavior and Ca^2+^ activity before (Pre) and 5 and 15 min after oxycodone. **(A)** When comparing Pre, 5 and 15 min after oxycodone injection there was a statistically significant increase in distance traveled among groups (one-way RM ANOVA *F*_2,14_ = 8.14, *p* = 0.005), with Pre *versus* 5 min (*p* = 0.005) and pre *versus* 15 min (*p* = 0.028) significantly increasing, but 5 min *versus* 15 min (*p* = 0.63) showing no difference. When comparing Pre, 5 and 15 min after oxycodone injection there was a statistically significant increase in velocity among groups (one-way RM ANOVA *F*_2,14_ = 8.97, *p* = 0.003), with pre *versus* 5 min (*p* = 0.004) and pre *versus* 15 min (*p* = 0.017) significantly increasing, but 5 min *versus* 15 min (*p* = 0.71) showing no difference. **(B)** Ca^2+^ activity, as reflected by the number of active cells, showed a progressive decrease with time. There was a statistically significant decrease in total number of active cells in D1-Cre mice (one-way ANOVA, *F*_2,6_ = 6.39, *p* = 0.03) observed between Pre *versus* 15 min (*post-hoc* Tukey test, *p* = 0.04). There was a statistically significant decrease in the total number of active cells in A2A-Cre mice (one-way ANOVA, *F*_2,9_ = 13.50, *p* = 0.002) with a significant decrease observed between Pre *versus* 5 min and Pre *versus* 15 min (*post-hoc* Tukey test, *p* = 0.009 and *p* = 0.002, respectively). When combining D1-Cre and A2A-Cre data there was a statistically significant decrease in total number of active cells (one-way ANOVA *F*_2,18_ = 18.8, *p* < 0.001) with a significant decrease observed between Pre *versus* 5 min and Pre *versus* 15 min (*post-hoc* Tukey test, *p* < 0.001). **p* < 0.05, ^**^*p* < 0.01, ^***^*p* < 0.001.

Supplementary Figure 3 Ca^2+^ transient frequency as a function of movement *versus* non-movement epochs. **(A)** In striatum, the frequency of Ca^2+^ transients before drug administration was consistently higher during movement compared with non-movement in D1- and A2A-Cre mice. After oxycodone, this relation persisted in D1- but not in A2A-Cre mice. **(B)** In the cerebral cortex, the correlation between behavior and Ca^2+^ transient frequency was less evident than in striatum but still could be observed. After oxycodone, this relationship disappeared. Data were analyzed using Student’s *t*-test, **p* < 0.05.

Supplementary Figure 4 **(A)** Traces represent multi-neuronal Ca^2+^ transients recorded in an A2A-Cre mouse before and 15 min after saline IP injection. Each trace represents the activity of one medium-sized spiny neuron (MSN). The number of active cells is similar before and after saline. **(B)** Upward deflections indicate the mouse was moving. In both conditions the behavior appears random and is tightly correlated with Ca^2+^ transients. **(C)** Some representative Ca^2+^ transient traces of single MSNs. **(D)** Bar graphs show the average frequency of Ca^2+^ transients before and after saline IP injection. Saline injections did not affect Ca^2+^ transient frequency in grouped MSNs (*p* = 0.53, Student’s *t*-test).

Supplementary Figure 5 Traces exemplify one medium-sized spiny neuron (MSN) from D1-Cre or A2A-Cre mice and one CPN from Syn or CaMKII mice before and 15 min after oxycodone. In striatum, the same MSN showed reduced activity, whereas in cortex the same CPN displayed increased activity.

Supplementary Video 1 Raw Miniscope video of Ca^2+^ activity before (Pre) oxycodone in a D1-Cre mouse injected with floxed GCaMP6f.

Supplementary Video 2 Raw Miniscope video of Ca^2+^ activity 15 min after oxycodone in a D1-Cre mouse injected with floxed GCaMP6f.

Supplementary Video 3 Raw Miniscope video of Ca^2+^ activity before (Pre) oxycodone in an A2A-Cre mouse injected with floxed GCaMP6f.

Supplementary Video 4 Raw Miniscope video of Ca^2+^ activity 15 min after oxycodone in an A2A-Cre mouse injected with floxed GCaMP6f.

Supplementary Video 5 Raw Miniscope video of Ca^2+^ activity before (Pre) oxycodone in a mouse injected with Syn-GCaMP6f.

Supplementary Video 6 Raw Miniscope video of Ca^2+^ activity 15 min after oxycodone in a mouse injected with Syn-GCaMP6f.

Supplementary Video 7 Two-photon laser-scanning microscopy (2PLSM) video of Ca^2+^ activity before (Pre) oxycodone, in the presence of 4-AP (100 μM), in a D1-Cre mouse used for Miniscope recordings.

Supplementary Video 8 Two-photon laser-scanning microscopy (2PLSM) video of Ca^2+^ activity after oxycodone application, in the presence of 4-AP (100 μM), in a D1-Cre mouse used for Miniscope recordings.
