# Supplementary figures and images for: Dissociable effects of oxycodone on behavior, calcium transient activity, and excitability of dorsolateral striatal neurons
Source: Front Neural Circuits. 2022 Oct 26;16:983323. doi: 10.3389/fncir.2022.983323 (PMC9643681; doi:10.3389/fncir.2022.983323)

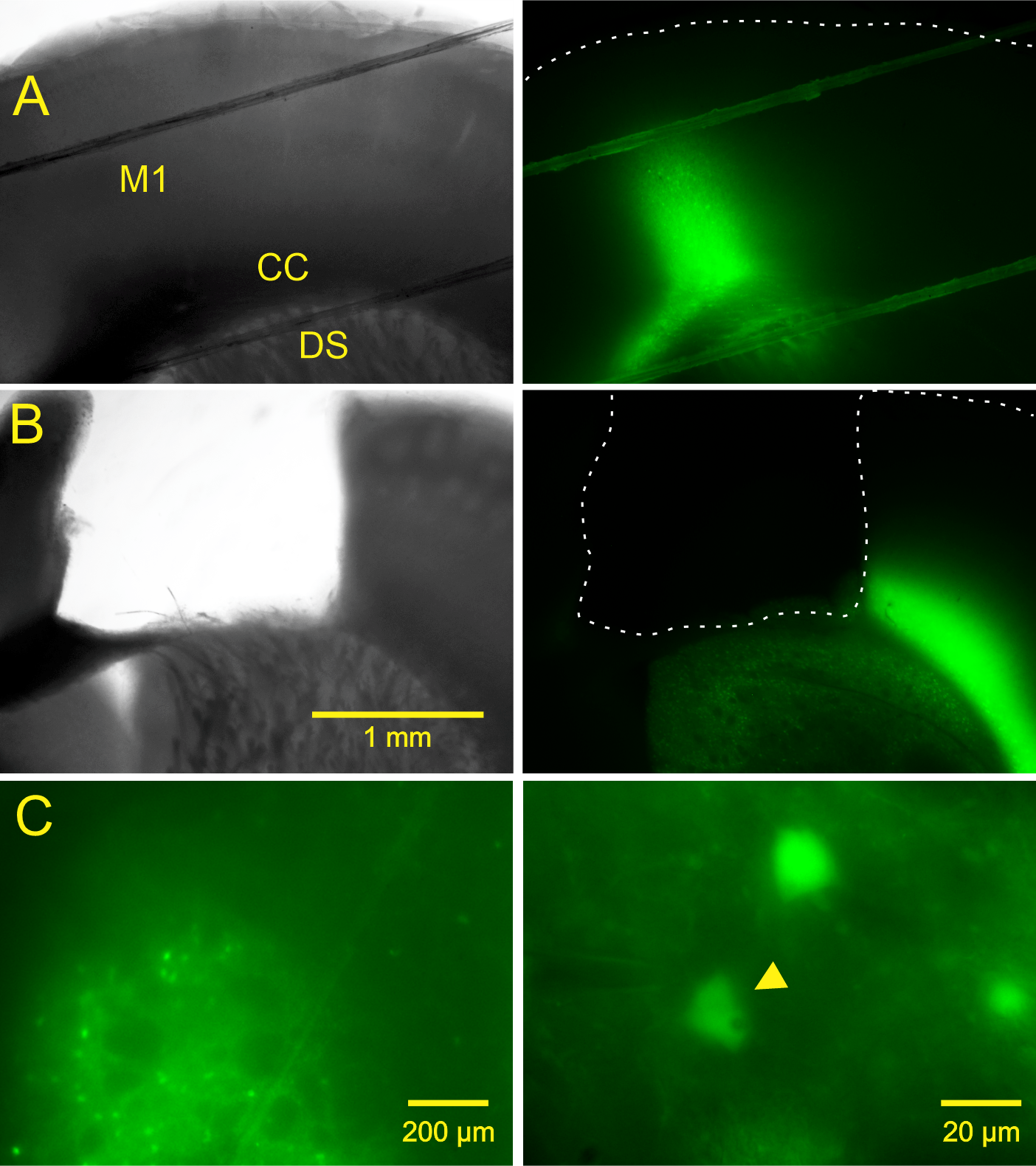

Supplement: Supplementary Figure 1 — Example slices (350 μm) obtained from mice used for Miniscope recordings. (A) Cortical slice showing the site and spread of viral injection (CaMKII promoter) under infrared DIC optics (left panel) and under fluorescence microscopy (right panel). The injection corresponds to M1 motor cortex. (B) Example slice obtained from a D1-Cre mouse used for striatal Miniscope recordings. Left panel shows the extent of cortical tissue aspirated for GRIN lens implant. The spread of the viral injection remained circumscribed to the striatum, although it was common to see fluorescence along the corpus callosum and in the tissue surrounding the GRIN lens. (C) Higher magnification of a striatal slice from a D1-Cre mouse used for Miniscope recordings, but on the intact (non-aspirated) side. This slice was used for electrophysiological recordings of identified medium-sized spiny neurons (MSNs). The cell indicated by the yellow arrow was patched and the recording is shown in Figure 6A. [file Image_1.TIF]

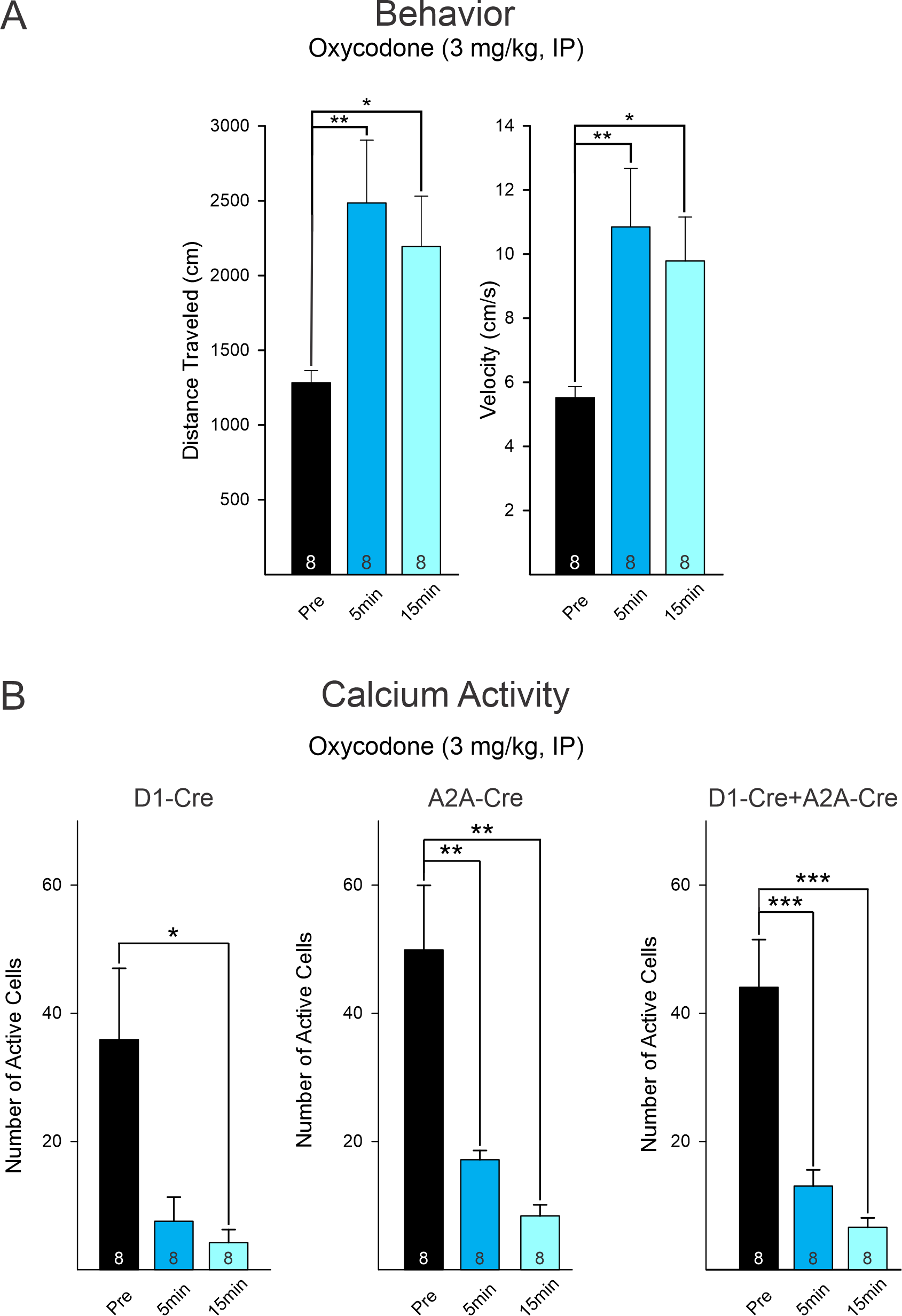

Supplement: Supplementary Figure 2 — To determine the time course of oxycodone effects we examined behavior and Ca2+ activity before (Pre) and 5 and 15 min after oxycodone. (A) When comparing Pre, 5 and 15 min after oxycodone injection there was a statistically significant increase in distance traveled among groups (one-way RM ANOVA F2,14 = 8.14, p = 0.005), with Pre versus 5 min (p = 0.005) and pre versus 15 min (p = 0.028) significantly increasing, but 5 min versus 15 min (p = 0.63) showing no difference. When comparing Pre, 5 and 15 min after oxycodone injection there was a statistically significant increase in velocity among groups (one-way RM ANOVA F2,14 = 8.97, p = 0.003), with pre versus 5 min (p = 0.004) and pre versus 15 min (p = 0.017) significantly increasing, but 5 min versus 15 min (p = 0.71) showing no difference. (B) Ca2+ activity, as reflected by the number of active cells, showed a progressive decrease with time. There was a statistically significant decrease in total number of active cells in D1-Cre mice (one-way ANOVA, F2,6 = 6.39, p = 0.03) observed between Pre versus 15 min (post-hoc Tukey test, p = 0.04). There was a statistically significant decrease in the total number of active cells in A2A-Cre mice (one-way ANOVA, F2,9 = 13.50, p = 0.002) with a significant decrease observed between Pre versus 5 min and Pre versus 15 min (post-hoc Tukey test, p = 0.009 and p = 0.002, respectively). When combining D1-Cre and A2A-Cre data there was a statistically significant decrease in total number of active cells (one-way ANOVA F2,18 = 18.8, p < 0.001) with a significant decrease observed between Pre versus 5 min and Pre versus 15 min (post-hoc Tukey test, p < 0.001). *p < 0.05, **p < 0.01, ***p < 0.001. [file Image_2.TIF]

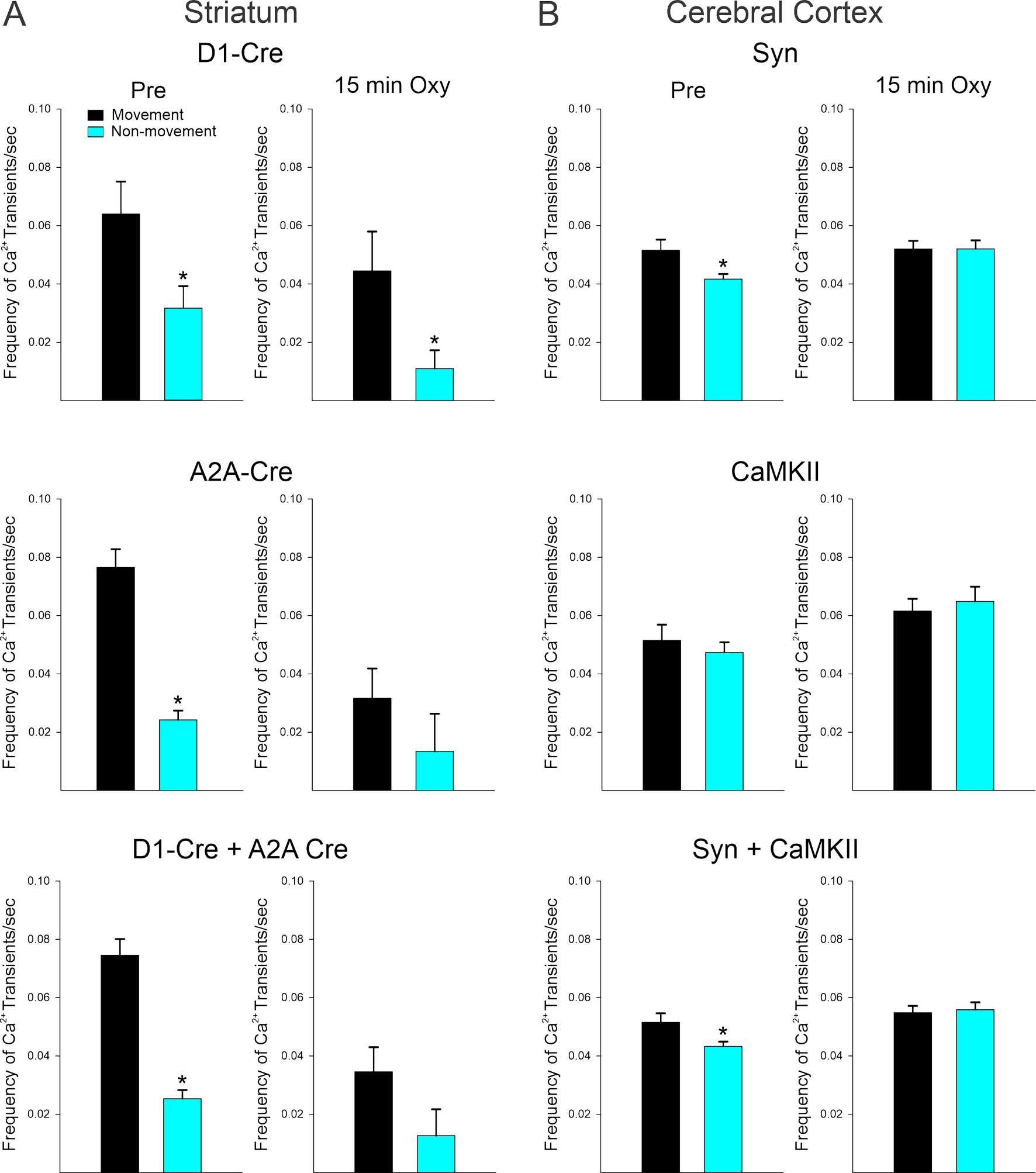

Supplement: Supplementary Figure 3 — Ca2+ transient frequency as a function of movement versus non-movement epochs. (A) In striatum, the frequency of Ca2+ transients before drug administration was consistently higher during movement compared with non-movement in D1- and A2A-Cre mice. After oxycodone, this relation persisted in D1- but not in A2A-Cre mice. (B) In the cerebral cortex, the correlation between behavior and Ca2+ transient frequency was less evident than in striatum but still could be observed. After oxycodone, this relationship disappeared. Data were analyzed using Student’s t-test, *p < 0.05. [file Image_3.TIF]

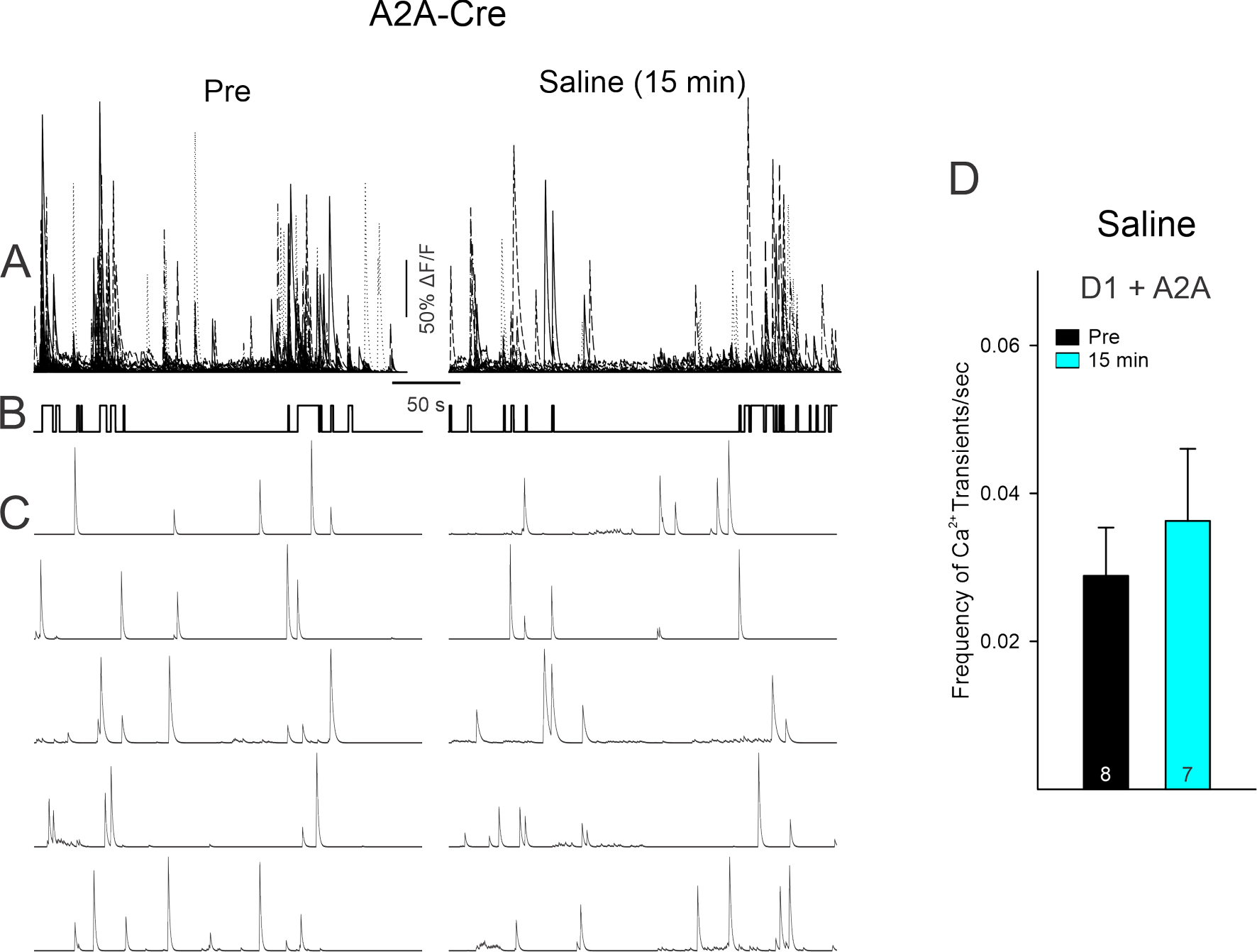

Supplement: Supplementary Figure 4 — (A) Traces represent multi-neuronal Ca2+ transients recorded in an A2A-Cre mouse before and 15 min after saline IP injection. Each trace represents the activity of one medium-sized spiny neuron (MSN). The number of active cells is similar before and after saline. (B) Upward deflections indicate the mouse was moving. In both conditions the behavior appears random and is tightly correlated with Ca2+ transients. (C) Some representative Ca2+ transient traces of single MSNs. (D) Bar graphs show the average frequency of Ca2+ transients before and after saline IP injection. Saline injections did not affect Ca2+ transient frequency in grouped MSNs (p = 0.53, Student’s t-test). [file Image_4.TIF]

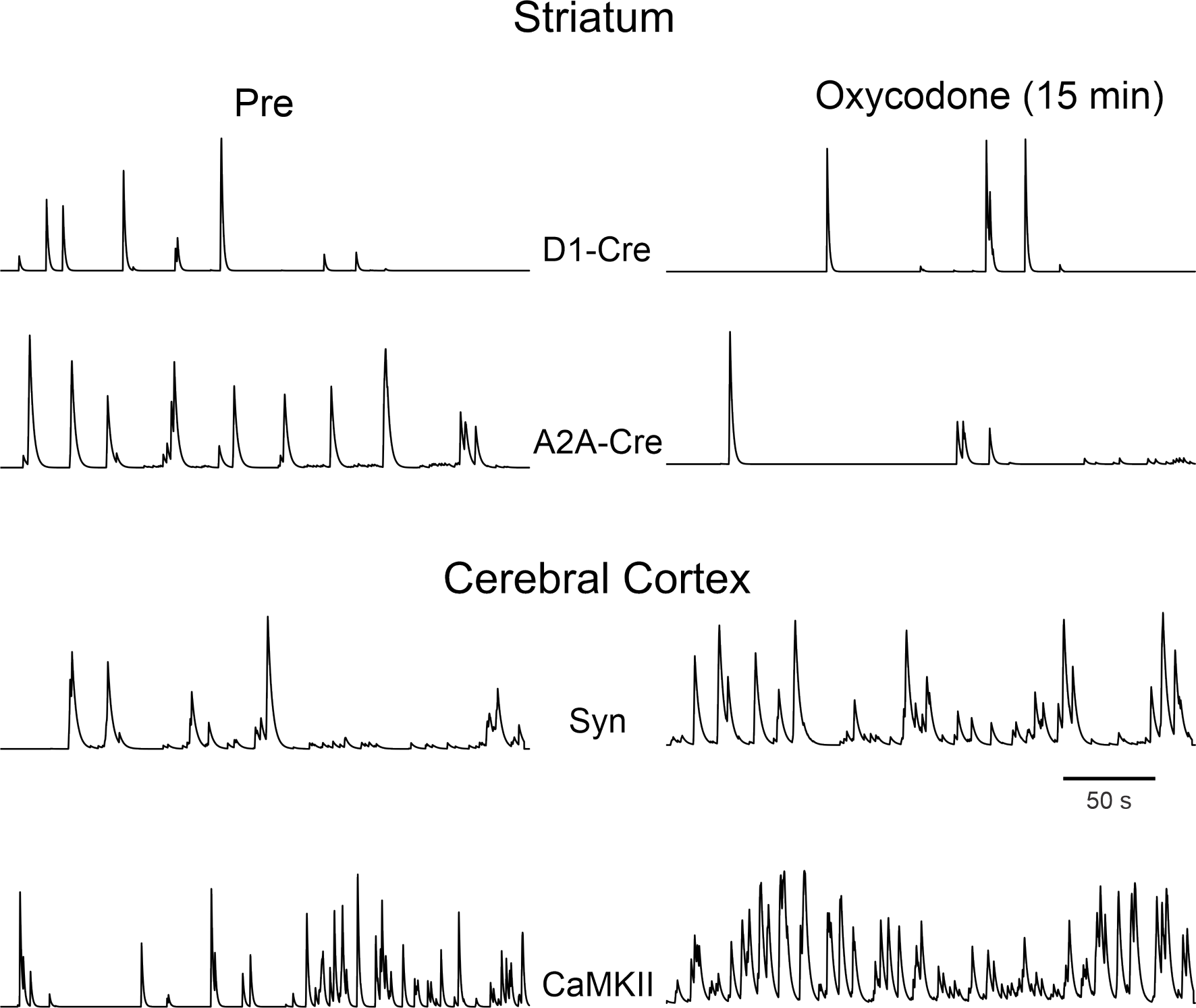

Supplement: Supplementary Figure 5 — Traces exemplify one medium-sized spiny neuron (MSN) from D1-Cre or A2A-Cre mice and one CPN from Syn or CaMKII mice before and 15 min after oxycodone. In striatum, the same MSN showed reduced activity, whereas in cortex the same CPN displayed increased activity. [file Image_5.TIF]
