# Supplementary material for: Dissociable effects of oxycodone on behavior, calcium transient activity, and excitability of dorsolateral striatal neurons
Source: Front Neural Circuits. 2022 Oct 26;16:983323. doi: 10.3389/fncir.2022.983323 (PMC9643681; doi:10.3389/fncir.2022.983323)
Supplement: Supplementary file 14 [file Table_1.DOCX]

**Supplement Table 1: Number of Mice, Videos, and Cells used for Miniscopes Data Analysis**

**# mice # videos # cells cells/video**

**Striatum (Pre and Oxycodone)**

D1-Cre 3 3 108 36

A2A-Cre 3 4 200 50

**Cortex (Pre and Oxycodone)**

Syn 2 3 387 129

CaMKII 2 2 156 78

**Striatum (Pre and Saline)**

D1-Cre 2 3 50 17

A2ACre 2 5 315 63

**Cortex (Pre and Saline)**

Syn 1 1 174 174
